# Association of a Prior Psychiatric Diagnosis With Mortality Among Hospitalized Patients With Coronavirus Disease 2019 (COVID-19) Infection

**DOI:** 10.1001/jamanetworkopen.2020.23282

**Published:** 2020-09-30

**Authors:** Luming Li, Fangyong Li, Frank Fortunati, John H. Krystal

**Affiliations:** 1 Yale University School of Medicine, Department of Psychiatry, New Haven, Connecticut; 2 Yale University School of Public Health, New Haven, Connecticut

## Abstract

This cohort study evaluates the association between prior psychiatric diagnosis and mortality among patients hospitalized with coronavirus disease 2019 (COVID-19).

## Introduction

Psychiatric disorders are associated with shortened life expectancy (ie, shortened by as much as 10 years).^1^ There is a concern that psychiatric comorbidity might increase Coronavirus Disease 2019 (COVID-19)–related mortality, as suggested by prior preliminary studies of cardiac and infectious disease outcomes.^2,3^ A large population study in Demark suggested that an a priori diagnosis of depression was associated with a higher 30-day mortality for those hospitalized for an infection.^3^ Here, we evaluate the association between having any prior psychiatric diagnosis and COVID-19–related mortality of hospitalized patients with COVID-19.

## Methods

This cohort study was conducted at Yale New Haven Health System, a 5-hospital system in the Northeast of the United States. Data were obtained from Epic Systems and included all encounters of hospitalized COVID-19–positive patients between February 15 and April 25, 2020, and followed up to May 27, 2020, for mortality. Descriptive statistics were used to characterize these patients with or without any prior psychiatric diagnoses. Kaplan-Meier analysis was conducted to compare the survival rates using the log-rank test. Univariate Cox proportional hazards regression was used to assess the association of pretreatment risk factors (including age, sex, race/ethnicity, medical comorbidities, and hospital location) with mortality. A multivariable Cox regression analysis was performed to confirm the association of psychiatric comorbidities with mortality after controlling for other risk factors. All analyses were conducted using SAS, version 9.4 (SAS Institute Inc), testing for a 2-sided significance level of .05. The study was approved for exemption by the institutional review board of Yale New Haven Health System, and a waiver of consent was granted. The study used deidentified data and was considered a medical record–only review by the institutional review board. The study followed the Strengthening the Reporting of Observational Studies in Epidemiology (STROBE) reporting guideline for cohort studies.

## Results

**Figure 1.**
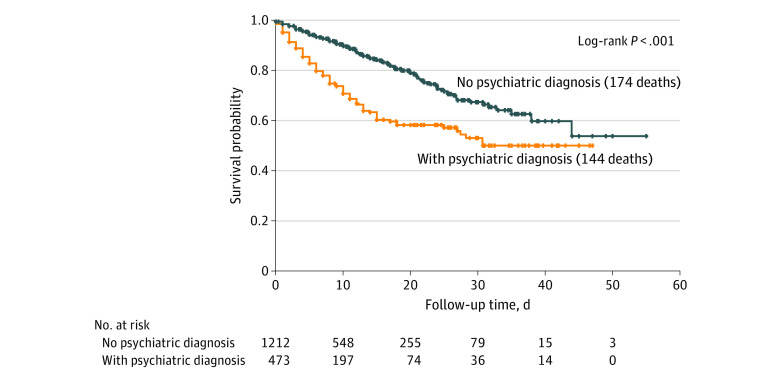
Kaplan-Meier Survival Curves for Hospitalized Patients With Coronavirus Disease 2019, With or Without a Psychiatric Diagnosis

**Figure 2.**
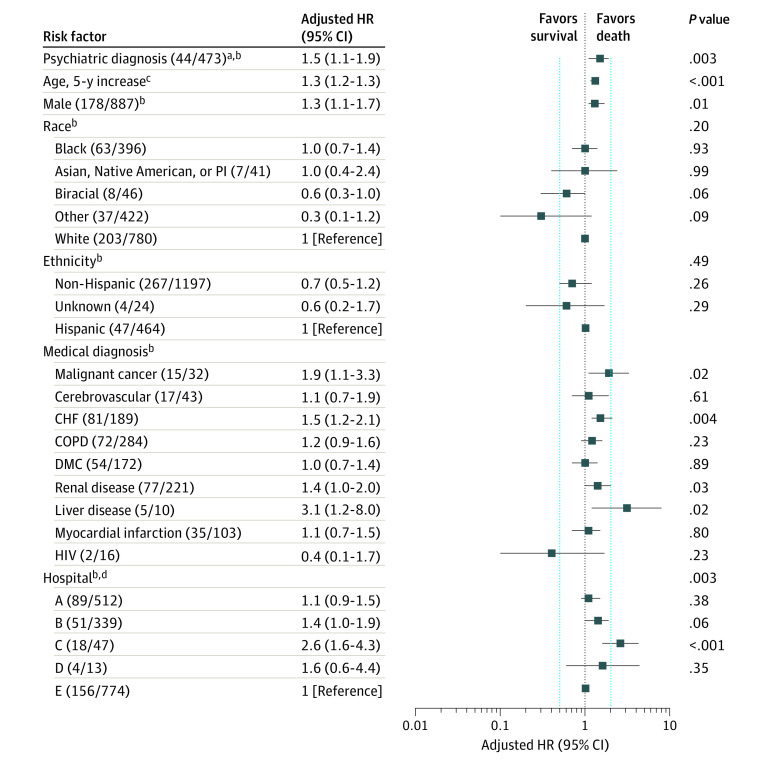
Multivariable Cox Hazard Regression Assessing Risk of Death for Hospitalized Patients With Coronavirus Disease 2019 (COVID-19) and Psychiatric Diagnosis CHF indicates congestive heart failure; COPD, chronic obstructive pulmonary disease; DMC, diabetes with complications; HR, hazard ratio; PI, Pacific Islander. ^a^Psychiatric diagnosis included *International Statistical Classification of Diseases and Related Health Problems, Tenth Revision* codes F01 to 99 (mental and behavioral health), specific codes G30 to 32 (Alzheimer disease), and codes X60 to 63 (self-injury). ^b^The numbers in each categorical variable correspond with the number of deaths in the numerator and the number of inpatients hospitalized with COVID-19 with that categorical variable in the denominator. ^c^The HR for age is presented as an increase of risk with every 5-year increase in age. ^d^Hospitals A, B, C, and D represent community hospitals affiliated with the health system, and Hospital E represents an academic medical center within the health system. None of the hospitals were rehabilitation hospitals. Although there are psychiatric units associated with the academic medical center and 2 of the community hospitals, the patients who were hospitalized for COVID-19 were hospitalized in the general hospitals for hospital-based care or intensive care.

A total of 1685 patients were hospitalized with COVID-19 during the study period (mean [SD] age, 65.2 [18.4] years; 887 [52.6%] were male). Of the 1685 patients, 473 (28%) received psychiatric diagnoses prior to hospitalization. Patients with psychiatric diagnoses were significantly older and more likely to be female, white, and non-Hispanic and have medical comorbidities (malignant cancer, cerebrovascular disease, congestive heart failure, diabetes, kidney disease, liver disease, myocardial infarction, and/or HIV). Overall, 318 patients (18.9%) died. Patients with a psychiatric diagnosis had a higher mortality rate compared with those with no psychiatric diagnosis (Figure 1), with 35.7% vs 14.7% of 2-week mortality and 40.9% vs 22.2% of 3-week mortality rate (*P* < .001) (and with 44.8% vs 31. 5% of 4-week mortality rate). The median follow-up time was 8 days (interquartile range, 4-16 days). In the unadjusted model, the risk for COVID-19–related hospital death was greater for those with any psychiatric diagnosis (hazard ratio, 2.3; 95% CI, 1.8-2.9; *P* < .001). After controlling for demographic characteristics, other medical comorbidities, and hospital location, the risk of death remained significantly greater among patients with a psychiatric disorder (hazard ratio, 1.5; 95% CI, 1.1-1.9; *P* = .003) (Figure 2).

## Discussion

This is the first study, to our knowledge, that characterizes the association of psychiatric diagnosis with COVID-19–related mortality. The primary finding is that patients with a prior psychiatric diagnosis while hospitalized for COVID-19 had a higher mortality rate compared those without a psychiatric condition. The finding is similar to previous findings: individuals with concurrent psychiatric and medical diagnoses had poorer outcomes and higher mortality.^4,5^

It is unclear why psychiatric illness predisposes to COVID-19–related mortality. Psychiatric symptoms may arise as a marker of systemic pathophysiologic processes, such as inflammation, that may, in turn, predispose to mortality. Similarly, psychiatric disorders may augment systemic inflammation and compromise the function of the immune system, while psychotropic medications may also be associated with to mortality risk.^6^

The limitations to the study include the fact that those individuals not hospitalized for COVID-19 or who died outside the hospital were not used in the analysis. In addition, diagnosis codes were used to assess for any psychiatric diagnosis, without accounting for the status of psychiatric treatment (patient with active, in-remission, or recovered psychiatric disorder). The data also do not include COVID-19 treatment information.
